# miRNA-494 in Lymphocytes: A Promising Biomarker for Acute Ischemic Stroke

**DOI:** 10.31083/RN37809

**Published:** 2025-08-29

**Authors:** Zixian Xie, Ziping Han, Tong Shen, Liyuan Zhong, Junfen Fan, Rongliang Wang, Feng Yan, Haiping Zhao, Qingfeng Ma, Yumin Luo

**Affiliations:** ^1^Institute of Cerebrovascular Diseases Research and Department of Neurology, Xuanwu Hospital of Capital Medical University, 100053 Beijing, China; ^2^Beijing Institute of Brain Disorders, Collaborative Innovation Center for Brain Disorders, Capital Medical University, 100069 Beijing, China

**Keywords:** microRNAs, ischemic stroke, lymphocytes, prognosis, microARN, ictus isquémico, linfocitos, pronóstico

## Abstract

**Background:**

microRNA-494 (miRNA-494) plays a key role in neuroinflammation following cerebral ischemia. We aimed to assess miRNA-494 levels as a biomarker for predicting acute ischemic stroke (AIS) severity and outcomes.

**Methods:**

miRNA-494 levels in peripheral lymphocytes were measured using reverse transcription-quantitative polymerase chain reaction. Least Absolute Shrinkage and Selection Operator (LASSO) regression was employed to identify variables for multivariate logistic regression analysis. Univariate and multivariate logistic regression were conducted to assess the association between miRNA-494 levels and both AIS outcomes and stroke severity on admission. The primary outcome was defined as an excellent prognosis (modified Rankin Scale score of 0 or 1). The secondary outcome was milder stroke severity at admission (National Institutes of Health Stroke Scale score <15).

**Results:**

High miRNA-494 expression in patients aged <65 years predicted excellent AIS outcomes (odds ratio (OR) = 2.800 [1.120–7.002], *p* = 0.028, n = 105). In these patients, miRNA-494 levels predicted excellent outcomes for those who did not receive recanalization therapy (continuous: OR = 8.938 [2.123–62.910], *p* = 0.010; categorical: OR = 5.200 [1.480–20.773], *p* = 0.013). Elevated miRN*A-494 *levels were also linked to milder stroke severity (continuous: OR = 2.586 [1.024–6.533], *p* = 0.044; categorical variables: OR = 3.514 [1.501–8.230], *p* = 0.004, n = 205).

**Conclusions:**

Increased miRNA-494 expression in lymphocytes predicts excellent outcomes in patients aged <65 years with AIS. Higher miRNA-494 levels are associated with milder stroke on admission.

## Abstract (es)

**Antecedentes:**

El microARN-494 (miARN-494) desempeña un papel clave en la neuroinflamación tras la isquemia cerebral. Nuestro objetivo fue evaluar los niveles de miARN-494 como biomarcador para predecir la gravedad y los resultados del ictus isquémico agudo (AIS, *acute ischemic stroke*).

**Métodos:**

Se midieron los niveles de miARN-494 en linfocitos periféricos mediante la reacción en cadena de la polimerasa cuantitativa con transcripción inversa. Se utilizó la regresión LASSO (Least Absolute Shrinkage and Selection Operator) para identificar las variables para el análisis de regresión logística multivariante. Se realizaron regresiones logísticas univariantes y multivariantes para evaluar la asociación entre los niveles de miARN-494 y los resultados del AIS y la gravedad del ictus al ingreso. El resultado principal se definió como un pronóstico excelente (puntuación de 0 o 1 en la escala de Rankin modificada). El resultado secundario fue una gravedad del ictus más leve al ingreso (puntuación en la escala del ictus de los Institutos Nacionales de Salud <15).

**Resultados:**

La expresión elevada de miARN-494 en pacientes menores de 65 años predijo resultados excelentes del AIS (cociente de posibilidades OR, *odds ratio* = 2,800 [1,120–7,002], *p* = 0,028, n = 105). En estos pacientes, los niveles de miARN-494 predijeron resultados excelentes para quienes no recibieron terapia de recanalización (continuo: OR = 8,938 [2,123–62,910], *p* = 0,010; categórica: OR = 5,200 [1,480–20,773], *p* = 0,013). Los niveles elevados de miARN-494 también se asociaron con una menor gravedad del ictus (continuo: OR = 2,586 [1,024–6,533], *p* = 0,044; variables categóricas: OR = 3,514 [1,501–8,230], *p* = 0,004, n = 205).

**Conclusiones:**

El aumento de la expresión de miARN-494 en los linfocitos predice excelentes resultados en pacientes menores de 65 años con AIS. Los niveles más altos de miARN-494 se asocian con un ictus más leve al ingreso.

## 1. Introduction

Cerebral ischemic stroke is one of the leading causes of mortality and disability worldwide [1, 2]. Currently, approximately half of patients with acute ischemic stroke (AIS) show no obvious abnormalities on computed tomography (CT) or magnetic resonance imaging (MRI) [3]. Some patients exhibit only atypical symptoms and commonly used assessment tools, such as the National Institutes of Health Stroke Scale (NIHSS), Glasgow Coma Scale, and modified Rankin scale (mRS), are often insufficient for accurate evaluation [4]. Consequently, the precise diagnosis of AIS remains an urgent challenge. Even among patients who receive recanalization therapy, only approximately one-third achieve excellent outcomes after 3 months [5]. Therefore, there is a pressing need to identify a reliable indicator to predict AIS prognosis and aid in more timely decision-making.

In recent years, non-coding RNAs, especially microRNAs (miRNAs), have garnered significant attention in biomarker research [6]. miRNAs are small non-coding RNA molecules (about 17–25 nucleotides) that help control gene expression by turning it down at the post-transcriptional level [7]. miRNA-mediated regulation of gene expression represents a key epigenetic mechanism in the nervous system [8]. After AIS, 24 types of miRNAs in endothelial progenitor cells were correlated with subacute stroke process and prognosis [9]. In addition to the cytoplasm and nucleus, miRNA and messenger RNA (mRNA) enrichment in mitochondria following cerebral ischemia or hypoxia can contribute to ischemia-reperfusion injury [10]. Growing evidence suggests that miRNAs may serve as important biomarkers for the diagnosis and prognosis of AIS and as potential therapeutic targets [11]. For instance, Sonoda *et al*. [7] identified seven serum miRNAs that could predict AIS risk before its onset.

Among these promising miRNAs, miRNA-494 has been associated with ischemic and neurodegenerative diseases [12]. In endothelial cells, miRNA-494 pretreatment reduces endoplasmic reticulum stress and increases cell viability [13]. In cardiomyocytes, miRNA-494 inhibits vascular smooth muscle apoptosis under oxidative stress, thereby enhancing cardio-protection following estrogen treatment [14, 15]. miRNA-494 is thought to target both pro-apoptotic and anti-apoptotic proteins, ultimately activating the protein kinase B (Akt) pathway, which offers protection against ischemia/reperfusion (I/R) damage in the heart [16]. However, the specific pro-inflammatory mechanisms related to AIS remain unclear. Immune cells, such as lymphocytes and neutrophils, can infiltrate the central nervous system and worsen I/R injury [17]. Our laboratory findings revealed that intravenous miRNA-494 antagonists exacerbate acute cerebral ischemic injury, modulate the expression of multiple matrix metalloproteinase subtypes, and influence neutrophil infiltration during the post-stroke reperfusion phase, partly by targeting histone deacetylase 2 (HDAC2). Additionally, miRNA-494 antagonism inhibits Th1 cell transition-related neurotoxicity [18, 19, 20].

Despite this evidence, the expression of miRNA-494 in patients with AIS and its potential clinical applications have not yet been explored. In this study, we aimed to detect miRNA-494 levels in the peripheral blood lymphocytes of patients with AIS and assess its potential as a biomarker of stroke severity and clinical outcomes predictions.

## 2. Materials and Methods

### 2.1 Study Participants

**Fig. 1.**
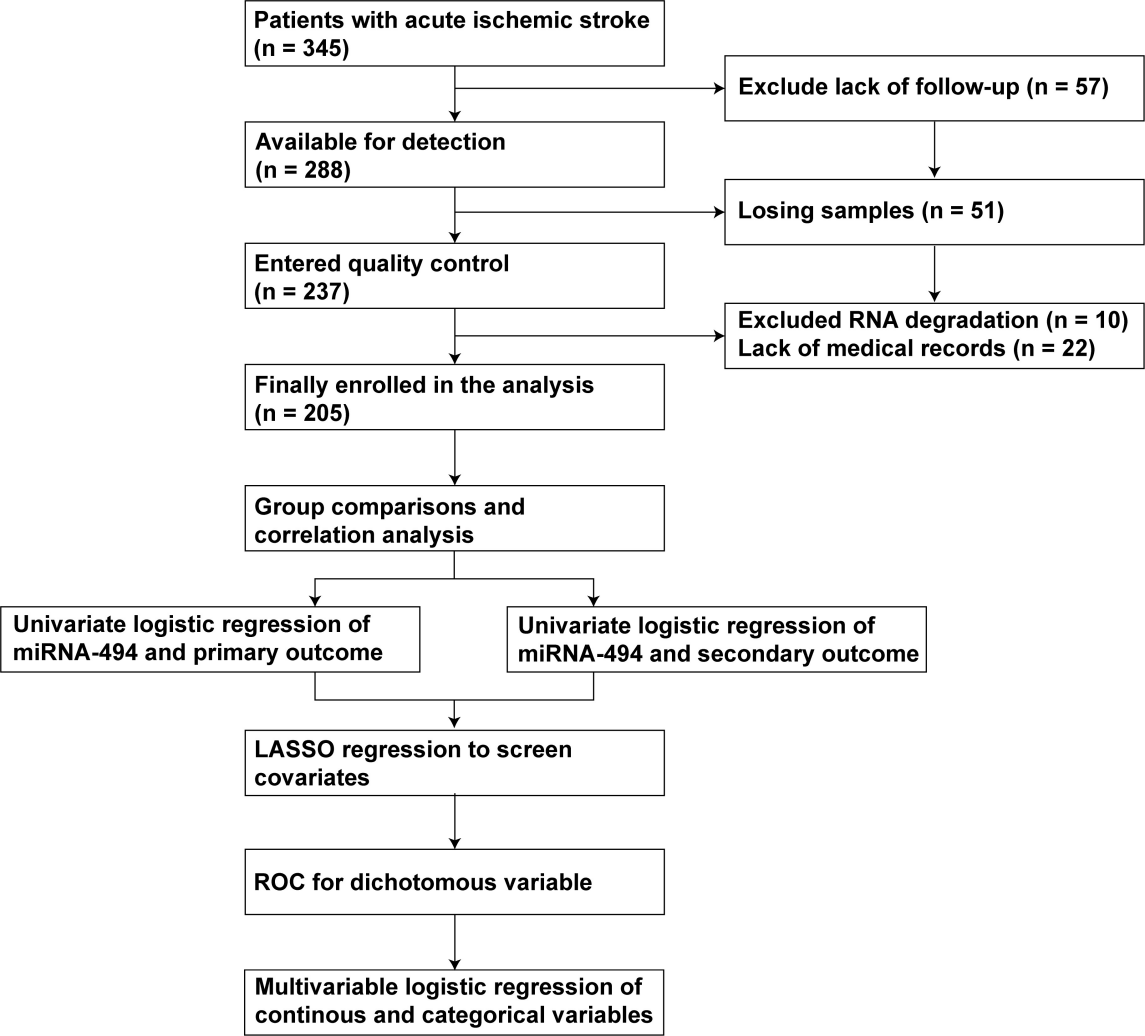
**Flow chart of this study**. Abbreviation: miRNA-494, microRNA-494; LASSO, least absolute shrinkage and selection operator; ROC, receiver operating characteristic.

The study was conducted in accordance with the Declaration of Helsinki and approved by the Institutional Review Board (or Ethics Committee) of Xuanwu Hospital, Capital Medical University. This study included 345 patients diagnosed with AIS by experienced neurologists at the Emergency and Neurology Department of Xuanwu Hospital between November 2018 and September 2019, as well as 37 healthy volunteers. The time elapsed from symptom onset to hospital admission was also confirmed by experienced physicians through detailed medical history taking. This duration will be referred to as “time to hospital admission” and “onset-to-treatment time” in the following text. Inclusion criteria for AIS patients were: (1) diagnosis confirmed via brain MRI or CT; (2) evident neurological deficits (such as motor and/or sensory deficits on the opposite side, impairment of higher cerebral functions and homonymous hemianopia); (3) age ≥18 years; (4) availability of complete case and follow-up data; (5) present within 24 h of symptom onset; and (6) consent to participate. The exclusion criteria were: (1) other cerebrovascular diseases such as cerebral hemorrhage diagnosed by CT or MRI, transient ischemic attack and hypertensive encephalopathy; (2) traumatic brain injury, encephalitis, multiple sclerosis or epilepsy; (3) severe infectious disease; (4) neoplasms; and (5) missing clinical baseline data. Ultimately, 205 patients with AIS were included in the regression model (Fig. 1).

Stroke severity was evaluated using the NIHSS, where scores 0–5 indicated minor stroke, 6–10 mild stroke, 11–14 moderate stroke, and 15–42 severe stroke [21, 22]. After 3 months, the mRS was used to assess patient outcomes. The primary outcome was defined as an excellent outcome (mRS score ≤1) at 3 months post-AIS onset [23, 24, 25, 26]. Secondary outcome was defined as minor-to-moderate stroke (NIHSS score <15) at admission [27].

According to the Trial of Org 10172 in Acute Stroke Treatment (TOAST) criteria [28], AIS patients were classified into four subgroups for descriptive and analytical purposes (no patients fell under “other determined etiology”): large-artery atherosclerosis, cardioembolic infarction, lacunar infarct and undetermined etiology.

Hypertension is defined as a clinic systolic blood pressure ≥140 mmHg and/or diastolic blood pressure ≥90 mmHg in the absence of antihypertensive medication [29]. Diabetes mellitus is defined as fasting plasma glucose ≥7.0 mmol/L and/or two-hour glucose ≥11.1 mmol/L after a 75 g oral glucose tolerance test based on optimal WHO criteria [30]. Hyperlipidemia (or dyslipidemia) is defined as the presence of one or more of the following in the serum: total cholesterol ≥6.2 mmol/L, triglycerides ≥2.3 mmol/L, low-density lipoprotein cholesterol ≥4.1 mmol/L, or high-density lipoprotein cholesterol <1.0 mmol/L [31].

### 2.2 Separation of Human Peripheral Lymphocytes

Peripheral venous blood from patients on admission and healthy volunteers was collected in ethylenediaminetetraacetic acid (EDTA) anticoagulant tubes. Lymphocytes were separated using the standard Ficoll-Paque Plus method. First, plasma and blood cells were separated through centrifugation. The blood cells were suspended in physiological saline and then carefully added along the wall of the centrifuge tube into the Lymphocyte Separation Medium (Haoyang Biotech, LTS1077, Tianjin, China). Following centrifugation, the lymphocytes from the middle layer were harvested and added to the red blood cell lysis solution (155 mM NH_4_Cl, 12 mM NaHCO_3_ and 0.1 mM EDTA, 12709101, 12600701, 10104201, Xilong Scientific Co., Ltd., Shantou, Guangdong, China). After vortexing and centrifugation, the pelleted cells were washed with physiological saline and transferred into new RNase-free EP tubes. The lymphocytes were finally suspended in 1 mL of TRIzol (Invitrogen Life Technologies, 15596026CN, Carlsbad, CA, USA) and stored at –80 °C until use. All samples were processed consistently throughout the study.

The samples were removed from –80 °C and allowed to thaw on ice; 200 µL of chloroform (10006818, Sinopharm Chemical Reagent Co., Ltd., Shanghai, China) was added to each EP tube, followed by vigorous shaking for 15 seconds and incubation at 25 °C for 15 minutes until phase separation was observed. The tubes were centrifuged (4 °C, 12,000 rpm, 15 minutes). Then, the upper aqueous phase was carefully transferred to a new RNase-free EP tube, and 500 µL of isopropanol (80109218, Sinopharm Chemical Reagent Co., Ltd., Shanghai, China) was added. The mixture was incubated overnight at –20 °C. The next day, the tubes were centrifuged again (4 °C, 12,000 rpm, 10 minutes). The supernatant was discarded, and the pellets were washed twice with 1 mL of 75% ethanol (10009218, Sinopharm Chemical Reagent Co., Ltd.). After removing the ethanol, the tubes were placed on a clean bench and air-dried at room temperature. Subsequently, 40 µL of diethylpyrocarbonate (DEPC)-treated water (Invitrogen Life Technologies, AM9920) was added to each tube to dissolve the RNA. The RNA purity and concentration were determined using a spectrophotometer, measuring absorbance ratios at 260/280 and 230/260. RNA integrity was assessed by agarose gel electrophoresis to check for any degradation.

### 2.3 Reverse Transcription Real-Time Quantitative Polymerase Chain Reaction (RT-qPCR)

The level of miRNA-494 in the peripheral blood lymphocytes of patients and volunteers was measured using RT-qPCR. First, cDNA was synthesized from total RNA using M-MuLV Reverse Transcriptase (P7040L; Enzymatics, Beijing, China). RT-qPCR was performed with a 2X PCR master mix (AS-MR-006-5, Arraystar, Rockville, MD, USA) on a QuantStudio5 Real-time PCR System (Applied Biosystems, Foster City, CA, USA). The PCR program was set as follows: initial denaturation at 95 °C for 10 minutes, followed by 40 cycles of PCR (95 °C for 10 seconds, 60 °C for 60 seconds with fluorescence collection). The mRNA levels were normalized to the level of *U6*, and the relative expression of each mRNA was calculated using the 2^-Δ⁢Δ⁢CT^ method. The following primers were used: *U6*, F: 5^′^ GCTTCGGCAGCACATATACTAAAAT 3^′^ and R: 5^′^ CGCTTCACGAATTTGCGTGTCAT 3^′^; has-miR-494-5p, GSP: 5^′^ GGGAGGTTGTCCGTGTTGT 3^′^ and R: 5^′^ GTGCGTGTCGTGGAGTCG 3^′^.

### 2.4 Statistical Analysis

All data were analyzed using R software (version 4.2.3, R Core Team, R Foundation for Statistical Computing, Vienna, Austria), IBM SPSS Statistics 25 (SPSS Inc., Chicago, IL, USA), and GraphPad Prism 8.2.1 (GraphPad Software, La Jolla, CA, USA). The criteria for type one error was set at α = 0.05. We assessed the normality of the data distribution using the Shapiro–Wilk test for samples with n < 50 or the Kolmogorov–Smirnov test for samples with n ≥ 50. For normally distributed data, the central tendency of continuous variables is described by the mean, with the standard deviation (SD) expressing the variability. For non-normally distributed data, the central tendency is represented by the median and the variability by the interquartile range. Categorical variables are presented as proportions. Depending on the data distribution, continuous variables were analyzed using either the Student’s *t*-test or the Mann–Whitney U test. Categorical variables were analyzed using either Pearson’s chi-square test or Fisher’s exact test. A logistic regression model was employed to determine the odds ratios (ORs) and 95% confidence intervals (95% CIs) to assess the association between miRNA-494 levels in lymphocytes and patient outcome at 3 months. This same analysis was conducted to evaluate the relationship between miRNA-494 and disease severity on admission. The receiver operating characteristic (ROC) curve and Youden index were used to convert miRNA-494 into a dichotomous variable [32]. Five-fold cross-validation of least absolute shrinkage and selection operator (LASSO) regression was used to screen valid clinical indicators. Sensitivity analysis was performed using multiple adjusted multivariate logistic regression models to test the robustness of miRNA-494 findings [33, 34]. For the prediction model linking miRNA-494 findings and outcomes, we included the admission NIHSS score, admission mRS score, systolic blood pressure (SBP), low-density lipoprotein cholesterol (LDL) level, and platelet count (PLT) as inclusion indicators for the follow-up logistic regression model. For the model relating miRNA-494 to disease severity on admission, we added the neutrophil-to-lymphocyte ratio (NLR), PLT on admission, history of coronary heart disease, and atrial fibrillation to the regression model. The variance inflation factor (VIF) is used to measure the severity of multicollinearity in a multiple linear regression model. When the VIF ≤10, it is considered that there is no multicollinearity.

## 3. Results

### 3.1 High Levels of miRNA-494 in Peripheral Lymphocytes May Have a Protective Effect in Patients With AIS

**Fig. 2.**
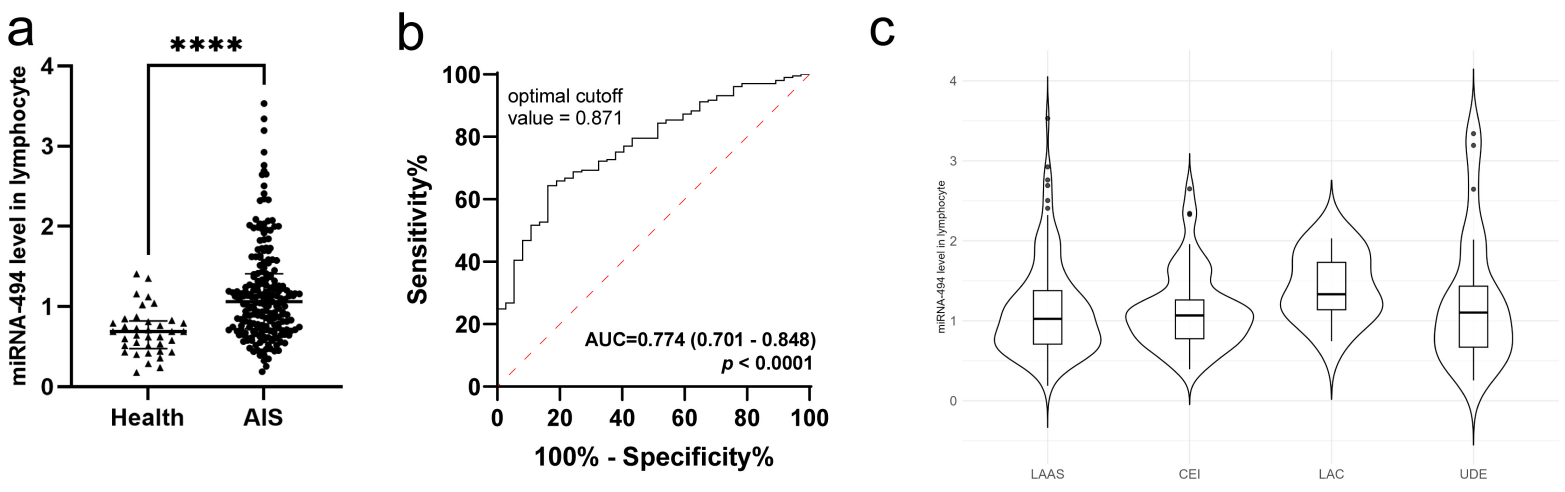
**miRNA-494 level was elevated in patients with AIS**. (a) Comparison between healthy volunteers and AIS patients, using Mann-Whitney U test (****, *p* < 0.0001), bars mean median and interquartile range. (b) ROC curve for miRNA-494 in lymphocytes to predict AIS, healthy group (n = 37), AIS group (n = 205). (c) miRNA-494 expression in different subtypes of AIS. Abbreviation: AIS, acute ischemic stroke; AUC, area under curve; LAAS, large artery atherosclerosis; CEI, cardioembolic infarct; LAC, lacunar infarct; UDE, undetermined etiology.

Initially, 205 patients with AIS and 37 healthy volunteers were analyzed for differences in miRNA-494 RNA levels in peripheral lymphocytes. The results indicated significantly higher expression in patients with AIS than in healthy individuals (Fig. 2a, *p* < 0.001). The ROC curve demonstrated the predictive ability of miRNA-494 levels in lymphocytes for diagnosing AIS. The area under the curve (AUC) for miRNA-494 levels predicting AIS was 0.774, with a 95% confidence interval of 0.701–0.848 (*p* < 0.0001) (Fig. 2b). The miRNA-494 levels in lymphocytes were also analyzed across different stroke subtypes. As shown in Fig. 2c and Table 1, no significant statistical differences were observed among the groups (*p* = 0.218, Kruskal-Wallis).

**Table 1.** **Baseline characteristics of the study population according to the outcome of patients at 3 months**.

		Total (N = 205)	Excellent outcome (N = 109)	Poor outcome (N = 96)	*p*
Demographic characteristics					
	Age (year, ± SD)	64.60 ± 13.31	61.53 ± 12.26	68.07 ± 13.65	<0.001
	Sex (%)	54 (26.34%)	27 (24.77%)	27 (28.12%)	0.700
Medical history					
	Hypertension	136 (66.34%)	67 (61.47%)	69 (71.88%)	0.154
	Diabetes mellitus	68 (33.17%)	32 (29.36%)	36 (37.50%)	0.277
	Coronary heart disease	43 (20.98%)	16 (14.68%)	27 (28.12%)	0.029
	Atrial fibrillation	36 (17.56%)	13 (11.93%)	23 (23.96%)	0.038
	Hyperlipidemia	60 (29.27%)	38 (34.86%)	22 (22.92%)	0.085
Clinical and laboratory finding					
	Onset-to-treatment time, h [IQR]	2.90 [1.60–5.10]	2.70 [1.40–4.30]	3.25 [1.85–6.35]	0.060
	Systolic blood pressure, mmHg [IQR]	150.00 [138.00–167.00]	150.00 [137.00–167.00]	150.00 [140.00–166.50]	0.995
	Diastolic blood pressure, mmHg [IQR]	85.00 [77.00–91.00]	85.00 [78.00–92.00]	84.50 [75.00–90.00]	0.312
	White blood cell count, ×1000/mm^3^ [IQR]	7.38 [6.00–8.84]	7.22 [6.17–8.83]	7.70 [5.99–8.84]	0.668
	Neutrophil count, ×1000/mm^3^ [IQR]	5.06 [3.88–6.45]	4.84 [3.85–5.92]	5.54 [3.89–7.11]	0.229
	Lymphocyte count, ×1000/mm^3^ [IQR]	1.53 [1.15–2.15]	1.74 [1.24–2.20]	1.42 [0.96–1.96]	0.003
	NLR [IQR]	2.88 [2.10–5.50]	2.63 [2.03–4.34]	3.61 [2.14–6.93]	0.011
	Platelet count, ×1000/mm^3^ [IQR]	207.00 [170.00–242.00]	216.00 [182.00–257.00]	196.00 [160.50–230.50]	0.005
	Triglyceride [IQR]	1.48 [0.96–2.48]	1.63 [1.00–2.70]	1.40 [0.89–2.07]	0.081
	Total cholesterol [SD]	4.53 [3.73–5.40]	4.65 [3.88–5.54]	4.43 [3.62–5.06]	0.063
	High density lipoprotein cholesterol [IQR]	1.19 [1.01–1.40]	1.17 [1.00–1.44]	1.20 [1.02–1.37]	0.910
	Low density lipoprotein cholesterol [IQR]	2.69 [2.03–3.41]	2.79 [2.10–3.54]	2.50 [1.99–3.30]	0.145
Treatment and measurement					
	NIHSS on admission [IQR]	6.00 [3.00–11.00]	3.00 [2.00–6.00]	10.00 [6.00–14.00]	<0.001
	mRS on admission [IQR]	3.00 [2.00–4.00]	2.00 [1.00–3.00]	4.00 [3.00–4.00]	<0.001
	Thrombolysis (%)	90 (43.90%)	53 (48.62%)	37 (38.54%)	0.190
	Thrombectomy (%)	32 (15.61%)	10 (9.17%)	22 (22.92%)	0.012
Etiology					
	Large artery atherosclerosis (LAAS)	116 (56.59%)	63 (57.80%)	53 (55.21%)	0.154
	Cardioembolic infarct (CEI)	54 (26.34%)	33 (30.28%)	21 (21.88%)	0.154
	Lacunar infarct (LAC)	10 (4.88%)	3 (2.75%)	7 (7.29%)	0.154
	Stroke of undetermined etiology (UDE)	25 (12.20%)	10 (9.17%)	15 (15.62%)	0.154

Excellent outcome: mRS score 0 to 1 at 3 months after AIS onset. Poor outcome: mRS score >1 at 3 months after AIS onset. Abbreviation: SD, standard deviation; IQR, Interquartile range; NLR, neutrophils to lymphocytes ratio; mRS, modified Rankin Scale; NIHSS, National Institutes of Health Stroke Scale.

After excluding patients with missing clinical indicators, we analyzed the baseline characteristics of the 205 patients, including demographics, past medical history, and various clinical indicators on admission, grouped by outcome (Table 1). The mean age of the patients was 64.60 ± 13.31 years, and 54 patients (26.34%) were female. The median NIHSS score on admission was 6, with a significant difference between the two outcome groups (*p* < 0.001). Regarding past medical history, significant differences were observed between the groups in patients with coronary heart disease (*p* = 0.029) and atrial fibrillation (*p* = 0.038) (Table 1).

Preliminary statistical analysis, including comparisons between the two outcome groups and correlation analysis, showed no significant correlation or association between miRNA-494 and the outcome at 3 months or with the mRS and NIHSS on admission. No trends were observed in the distribution plot of the miRNA-494 percentile divided by the interquartile range (**Supplementary Fig. 1a–f**). Additionally, miRNA-494 did not reach statistical significance in predicting the prognosis of all 205 patients using logistic regression (OR = 1.266, 95% CI: 0.794–2.018, *p* = 0.322).

**Fig. 3.**
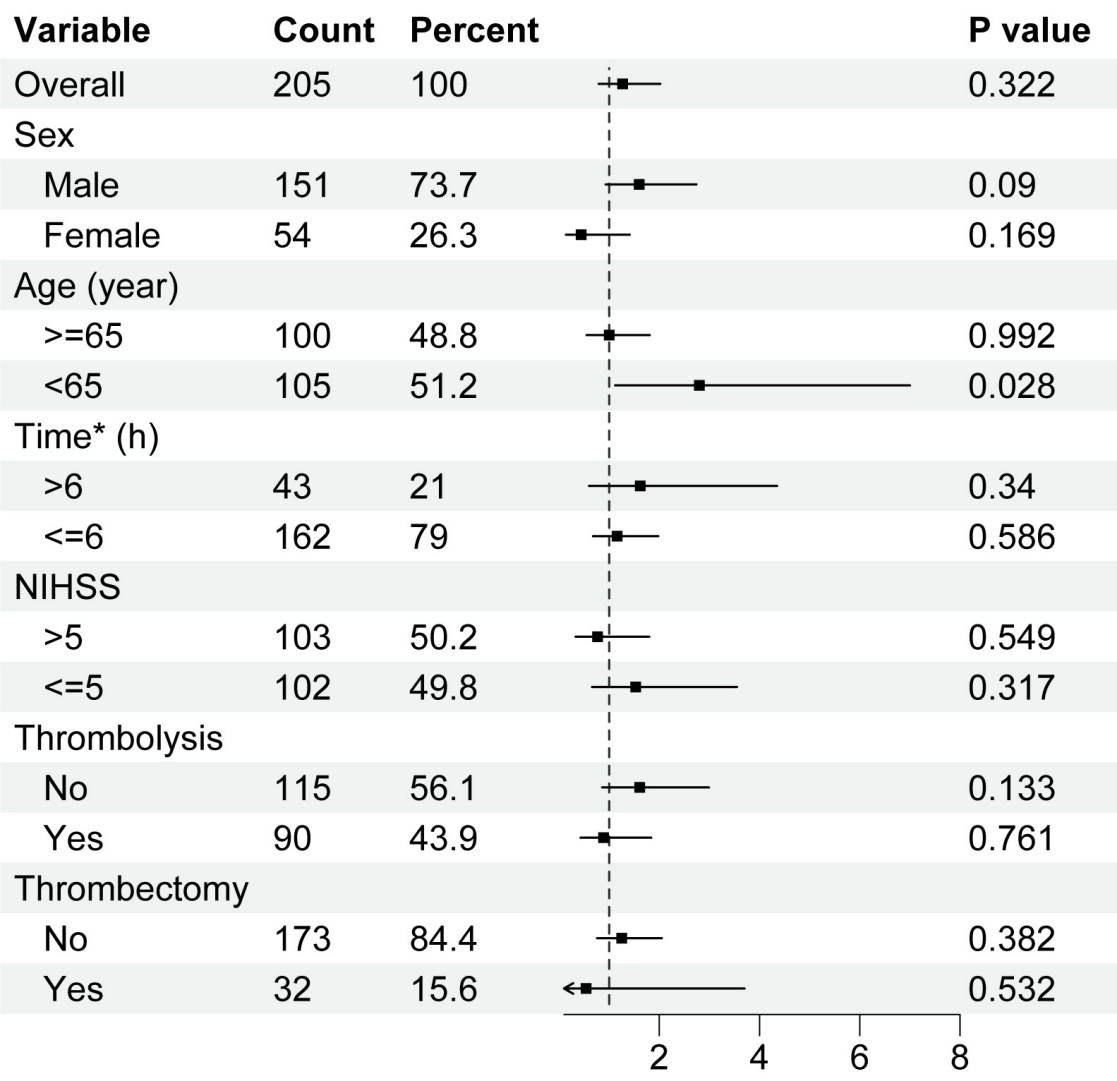
**Logistic regression analysis among subgroups of patients with acute ischemic stroke**. Univariate logistic regression on miRNA-494 levels and excellent outcome at 3 months of AIS patients, in each subgroup. Higher miRNA-494 levels in peripheral lymphocytes predicted better outcome. Horizontal axis was the level of miRNA-494. Abbreviation: NIHSS, National Institutes of Health Stroke Scale. *Time means onset-to-treatment time of patients.

To further explore whether miRNA-494 levels in peripheral lymphocytes predict AIS prognosis, we analyzed various subgroups. Univariate logistic regression was employed to calculate the OR, 95% CI, and *p*-values of miRNA-494 levels in each subgroup, including sex, age (cutoff of 65 years), time to hospital admission (cutoff of 6 h), NIHSS score (cutoff of 5), and treatment acceptance, such as thrombolysis and mechanical thrombectomy. These cutoff values were based on baseline statistical data and clinical considerations. The results indicated that only the group aged under 65 years demonstrated a statistically significant prognostic ability of miRNA-494 levels in peripheral lymphocytes (Fig. 3, *p* = 0.028).

### 3.2 Predictive Effect of miRNA-494 Levels in Lymphocytes on the Outcome at 3 Months After AIS in Patients Younger Than 65 Years

**Fig. 4.**
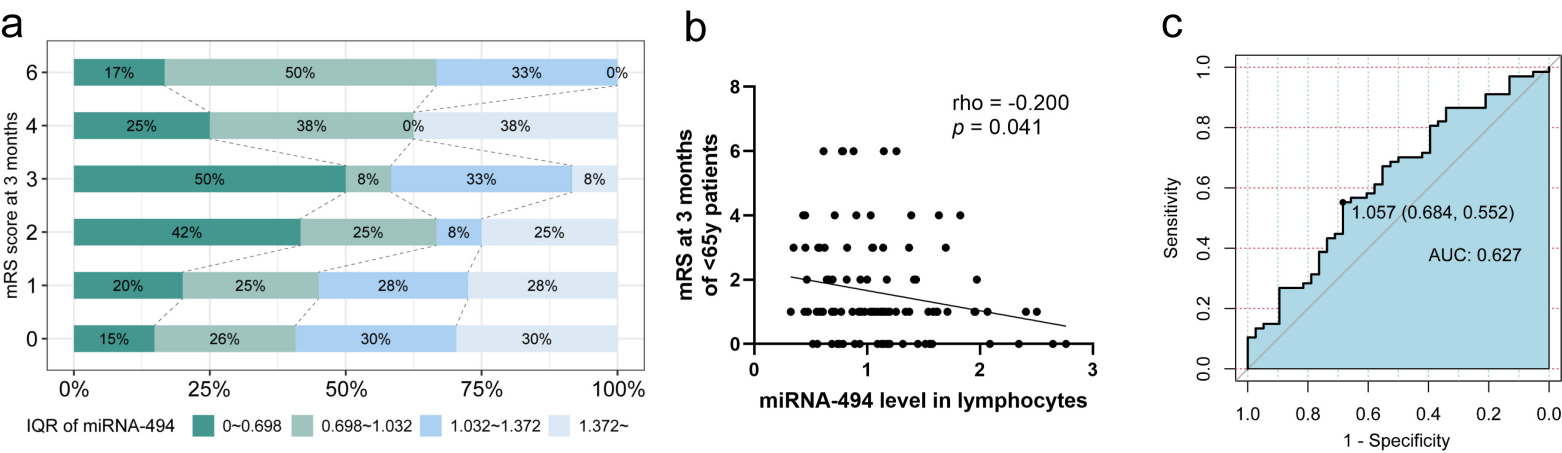
**Analysis of prognosis of patients with AIS aged under 65**. (a) Age <65 years old, distribution of miRNA-494 percentile among the 6 groups of mRS score at 3 months (n = 105). (b) Scatter plot and correlation, age <65 years old (n = 105), miRNA494 level was negatively correlated to mRS score after 3 months (*p* = 0.041). (c) ROC curve of miRNA-494 to predict 3 months outcome in age <65 years (n = 105). Abbreviation: mRS, modified Rankin Score.

We focused on patients aged under 65 years (n = 105). The distribution of miRNA-494 in lymphocytes among the six groups according to the mRS score at 3 months is shown in Fig. 4a, revealing a clear trend where lower mRS scores were associated with higher levels of miRNA-494. Additionally, miRNA-494 expression in lymphocytes was significantly negatively correlated with the mRS score at 3 months (Fig. 4b). Univariate logistic regression analysis indicated that the OR for miRNA-494 levels predicting an excellent outcome for patients 3 months post-stroke was 2.800 (95% CI: 1.120–7.002, *p* = 0.028) (Table 2). The ROC curve analysis identified the optimal cutoff point for miRNA-494 at 1.057, which yielded a sensitivity of 68.4% and specificity of 55.2% for AIS (Fig. 4c, AUC = 0.627, 95% CI: 0.516–0.739). This level was then categorized as a dichotomous variable with a cutoff point of 1.057. We assessed the association of miRNA-494 levels with AIS outcomes using univariate logistic regression analysis, revealing that miRNA-494 >1.057 independently predicted an excellent outcome at 3 months in patients younger than 65 years (Table 2, OR = 2.672 [95% CI: 1.158–6.168], *p* = 0.021).

**Table 2.** **miRNA-494 levels in lymphocytes and the excellent outcome of patients with age <65 years old (n = 105)**.

	Univariable model 1^*^			Multivariable model 2^&^			Multivariable model 3^⊚^		
	OR (95% CI)	R^2^	*p* value	OR (95% CI)	R^2^	*p* value	OR (95% CI)	R^2^	*p* value
miRNA-494^a^	2.800 (1.120–7.002)	0.052	0.028†	3.165 (1.222–8.199)	0.134	0.018†	2.565 (0.803–8.195)	0.332	0.112
miRNA-494 >1.057^b^	2.672 (1.158–6.168)	0.051	0.021†	2.788 (1.166–6.665)	0.125	0.021†	2.901 (1.031–8.159)	0.341	0.044†

^*^Model 1 was an unadjusted logistic regression model with the miRNA-494. ^&^Model 2 was an adjusted logistic regression model. The variables in model 2 included SBP, PLT and LDL on admission. ^⊚^Model 3 was an adjusted logistic regression model. The variables in model 3 included SBP, PLT, LDL, NIHSS score and mRS score on admission. ^a^ miRNA-494 as a continuous variable. ^b^ miRNA-494 as a categorical variable. †*p* < 0.05. Variance inflation factor of each valuable of different logistic models were all less than 10. Abbreviation: OR, odds ratio; CI, confidence interval; SBP, Systolic blood pressure; PLT, Platelet count; LDL, Low density lipoprotein cholesterol.

Following univariate analysis, we combined miRNA-494 levels with other clinical indicators to determine whether the predictive power remained reliable when accounting for additional factors. LASSO regression was employed to screen potential variables from 24 candidates, selecting miRNA-494 levels in lymphocytes, NIHSS score, mRS score on admission, SBP, PLT, and LDL as covariates for sensitivity analysis. As a continuous variable, miRNA-494 alone was statistically significant in predicting AIS prognosis, and the inclusion of SBP, PLT, and LDL did not significantly influence the results (Table 2, OR = 3.165 [95% CI: 1.222–8.199], *p* = 0.018). However, when including the NIHSS score, the predictive effect of miRNA-494 was less stable (Table 2, OR = 2.565 [95% CI: 0.803–8.195], *p* = 0.112). Nevertheless, as a categorical variable, miRNA-494 >1.057 not only maintained its independent predictive ability but also proved useful for predicting excellent outcomes when the model was adjusted for other clinical data (Table 2, OR = 2.901 [95% CI: 1.031–8.159], *p* = 0.044).

We further analyzed whether miRNA-494 and miRNA-494 >1.057 could serve as predictive factors in patients with or without recanalization therapy. In patients who did not undergo thrombolysis or mechanical thrombectomy, high levels of miRNA-494 significantly predicted excellent outcomes after 3 months, with OR values of 6.458 (95% CI: 1.871–31.922, *p* = 0.009), 3.449 (95% CI: 1.303–10.987, *p* = 0.022), and 8.938 (95% CI: 2.123–62.910, *p* = 0.010) (Table 3). Additionally, miRNA-494 >1.057 as a categorical variable also demonstrated predictive efficacy among patients not receiving either therapy (OR = 4.800 [95% CI: 1.551–16.234], *p* = 0.008; OR = 2.833 [95% CI: 1.147–7.439], *p* = 0.028; OR = 5.200 [95% CI: 1.480–20.733], *p* = 0.013) (Table 3). However, in patients who underwent either recanalization treatment, the univariate logistic regression analysis of miRNA-494 for the 3-month mRS did not yield significant results (Table 3).

**Table 3.** **miRNA-494 levels in lymphocytes and the excellent outcome of AIS patients age <65-year-old, divided by treatment**.

		miRNA-494^a^		miRNA-49*4* >1.057^b^	
		OR (95% CI)	*p* value	OR (95% CI)	*p* value
Overall (n = 105), 100%		2.800 (1.184–7.521)	0.028†	2.672 (1.177–6.328)	0.021†
Intravenous thrombolysis					
	Yes (n = 51), 48.6%	1.044 (0.260–4.591)	0.952	1.600 (0.467–6.019)	0.464
	No (n = 54), 51.4%	6.458 (1.871–31.922)	0.009†	4.800 (1.551–16.234)	0.008†
Endovascular thrombectomy					
	Yes (n = 13), 12.4%	0.529 (0.023–8.619)	0.656	2.500 (0.260–29.560)	0.433
	No (n = 92), 87.6%	3.449 (1.303–10.987)	0.022†	2.833 (1.147–7.439)	0.028†
Recanalization therapy					
	Either (n = 60), 57.1%	1.093 (0.306–4.127)	0.892	1.680 (0.564–5.302)	0.359
	Neither (n = 45), 42.9%	8.938 (2.123–62.910)	0.010†	5.200 (1.480–20.773)	0.013†

^a^ miRNA-494 as a continuous variable. ^b^ miRNA-494 as a categorical variable. †*p* < 0.05.

### 3.3 miRNA-494 Levels in Peripheral Lymphocytes Indicate Minor-to-Moderate Stroke

**Fig. 5.**
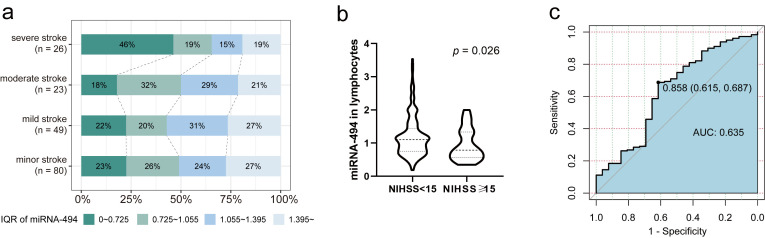
**Analysis of stroke-severity of patients with AIS**. (a) Division by interquartile range, under 4 classifications of severity according to NIHSS score on admission, the distribution of miRNA-494 levels percentiles of AIS patients (n = 205). (b) miRNA-494 level of two groups, cutoff by 15 score of NIHSS, Mann-Whitney U test, *p* = 0.026. (c) ROC curve of miRNA-494 levels to indicate NIHSS score on admission (n = 205). Abbreviation: ROC, receiver operating characteristic.

The relationship between miRNA-494 levels and admission NIHSS scores in 205 patients was analyzed using statistical methods and correlation analysis. In terms of severity, miRNA-494 levels showed a general downward trend in patients with more severe strokes (Fig. 5a). Based on the distribution of miRNA-494 levels and stroke severity, combined with clinical criteria and previous studies, the 205 patients with stroke were classified into minor-to-moderate and severe stroke groups, with an NIHSS score threshold of 15 [35, 36]. miRNA-494 levels in peripheral lymphocytes were significantly higher in patients with minor-to-moderate strokes compared with those with severe strokes (Fig. 5b, OR = 1.10 [95% CI: 0.74–1.42] vs. OR = 0.76 [95% CI: 0.53–1.29], *p* = 0.026). To establish a reliable association between miRNA levels and stroke severity, logistic regression and LASSO regression were applied to the 205 patients. miRNA-494 levels, coronary heart disease, atrial fibrillation history, PLT on admission, and NLR were identified as key variables. A multivariate logistic regression analysis using these variables showed that elevated miRNA-494 levels independently indicated minor-to-moderate stroke (Table 4, OR = 2.586 [95% CI: 1.024–6.533], *p* = 0.044).

**Table 4.** **miRNA-494 levels in lymphocytes and the severity of AIS patients (n = 205)**.

	Univariable model 1^*^			Multivariable model 2^#^			Multivariable model 3^&^		
	OR (95% CI)	R^2^	*p* value	OR (95% CI)	R^2^	*p* value	OR (95% CI)	R^2^	*p* value
miRNA-494 levels	2.586 (1.024–6.533)	0.024	0.044†	2.407 (0.926–6.261)	0.152	0.072	2.618 (0.955–7.177)	0.196	0.061
miRNA-494 >0.858	3.514 (1.501–8.230)	0.041	0.004†	3.346 (1.297– 8.631)	0.162	0.012†	3.881 (1.403–10.738)	0.207	0.009†

^*^Model 1 was an unadjusted logistic regression model with the miRNA-494. ^#^Model 2 was an adjusted logistic regression model. The variables in model 2 included miRNA-494, NLR, PLT on admission. ^&^Model 3 was an adjusted logistic regression model. The variables in model 3 included miRNA-494, NLR, PLT on admission, history of coronary heart disease, atrial fibrillation, hyperlipidemia. †*p* < 0.05. R^2^ was calculated using Cos and Snell way. Variance inflation factor of each valuable of different logistic models were all less than 10. Abbreviations: AIS, acute ischemic stroke; PLT, platelet count.

We also calculated the predictive value of miRNA-494 levels for minor-to-moderate strokes using ROC curve analysis. When miRNA-494 was treated as a dichotomous variable with a cutoff of 0.858, it had a sensitivity of 68.7% and a specificity of 61.5% for predicting minor-to-moderate stroke (Fig. 5c, AUC = 0.635 [0.786–0.615]). The association between dichotomous miRNA-494 levels and stroke severity was further evaluated using multivariate logistic regression. The OR for miRNA-494 levels >0.858 was 3.514 (1.501–8.230, *p *= 0.004), and after adjusting for other variables, miRNA-494 >0.858 remained a significant predictor of stroke severity (Table 4, OR = 3.881 [95% CI: 1.403–10.738], *p *= 0.009).

## 4. Discussion

Previous studies have shown that miRNA-494 mediates inflammation following stroke, suggesting that miRNA-494 may protect the nervous system by inhibiting Histone Deacetylase 3 (HDAC3) expression [18]. However, in neutrophils, antagonizing miRNA-494 reduced injury and neurotoxicity in experimental I/R models [19, 20]. In this study, miRNA-494 levels were higher in patients with AIS than in healthy individuals, consistent with previous findings [20]. Despite this, whether elevated miRNA-494 levels in lymphocytes are protective or a risk factor for AIS prognosis remains unclear. Insufficient statistical analysis has been conducted on the relationship between miRNA-494 and patient outcomes in AIS, whether in plasma, neutrophils, or lymphocytes. Therefore, we aimed to determine whether lymphocyte miRNA-494 could predict stroke prognosis.

Although miRNA-494 levels differed significantly between healthy volunteers and patients with AIS, they did not significantly predict excellent outcomes at 3 months among the 205 patients studied. One potential reason is that several confounding factors affecting miRNA-494 distribution were not controlled when enrolling patients. We did not exclude patients with immune system diseases, such as rheumatic heart disease, even though lymphocytes are closely tied to immune system functions. Another possible reason is that the time from symptom onset to hospital admission varies among patients, which leads to different time points for miRNA detection. The expression of certain miRNAs (such as miRNA-124, miRNA-210, miRNA-155, and miRNA-30a) has a nonlinear relationship with the duration of AIS illness [37]. However, whether miRNA-494 expression is time-dependent has not yet been studied. This variability may introduce bias into the statistical results. Therefore, we conducted subgroup analyses to determine whether other factors influenced miRNA-494’s predictive power.

Patients were classified as adults (age <65 years) or older adults (age ≥65 years). Among those under 65 years, high miRNA-494 levels in lymphocytes were reliable predictors of excellent outcomes. This may be explained by two factors: (1) In experimental stroke models, mice used are typically 2 months old, corresponding to young humans [38]. Therefore, experimental results may only apply to younger individuals. (2) Aging alters lymphocyte development and function, suggesting that miRNA-494 may play a more significant role in younger patients. The increasing incidence of ischemic stroke in young adults significantly elevates both medical and socioeconomic burdens, with long-lasting effects on their personal lives [36, 37]. Given the heterogeneous nature of stroke in younger individuals and the lack of sufficient studies on recurrence risks, it is crucial to individually assess and inform young patients about their specific risk of recurrent vascular events [39]. Moreover, therapeutic benefits for patients with stroke tend to be limited by age [40]. Therefore, this study may potentially offer new diagnostic and therapeutic insights for patients <65 years.

We also investigated the impact of recanalization therapy on miRNA-494’s predictive value. No significant relationship was found between miRNA-494 and outcomes in patients who underwent reperfusion treatment. However, miRNA-494 was a promising predictor of prognosis in patients under 65 years who did not receive recanalization therapy. We hypothesize that miRNA-494 in lymphocytes has a protective role against ischemia. However, this effect becomes ambiguous after brain reperfusion. These findings offer hope for patients who cannot undergo recanalization therapy within the narrow treatment window. However, the underlying mechanisms require further investigation.

A correlation between miRNA-494 and stroke severity was observed. Among all patients, higher levels of miRNA-494 (or miRNA-494 >0.858) were significantly associated with minor-to-moderate stroke. This may suggest that miRNA-494 upregulates in lymphocytes to protect against AIS, with higher levels correlating with milder strokes. However, miRNA-494 only ameliorated outcomes at 3 months in younger patients (<65 years), especially those who did not receive recanalization therapy.

The role of miRNA-494 in the nervous and immune systems remains contentious. Our analysis found no correlation between miRNA-494 levels in lymphocytes and lymphocyte or neutrophil counts (**Supplementary Fig. 1g,h**). In previous studies, miRNA-494 upregulation improved functional recovery, reduced injury, and inhibited apoptosis in rats after spinal cord injury (SCI) [41]. Similarly, in a hepatic I/R model, miRNA-494 reduced damage by inhibiting the Phosphatase and Tensin homologue/Phosphatidylinositol 3-kinase/Protein Kinase B (PTEN/PI3K/AKT) pathway [42]. Compared to healthy controls, miRNA-494 was upregulated in the peripheral blood of patients, likely as a protective response to acute-phase damage. Based on these studies, it is speculated that insufficient miRNA-494 upregulation in lymphocytes after I/R may result in weak protection. In neutrophils, miRNA-494 may act as a regulatory molecule that induces inflammation. Combined with the results of this study, low miRNA-494 expression in lymphocytes may lead to more severe stroke. Further research using experimental models is needed to elucidate the specific pathways involved in the protective mechanism of miRNA-494 in brain lymphocytes after I/R.

This study had certain limitations, including its single-center design, which restricts generalizability, and potential selection bias. The sample size of healthy controls is also relatively small (n = 37), which may affect one of the results, the higher expression of miRNA in the patient group compared to healthy individuals. Detailed clinical baseline information for these healthy volunteers was lacking, therefore, we could not further analyze between the two groups. Several risk factors associated with stroke, such as body mass index, alcohol or smoking habits, blood glucose levels, and stroke history, were not considered due to missing data. Additionally, the second part of the study focused on minor-to-moderate and severe strokes; however, only 26 patients (12.68%) had severe strokes. Future replication studies are necessary to further investigate the effects of miRNA-494 on AIS [43].

## 5. Conclusions

In summary, miRNA-494 levels in peripheral lymphocyte are significantly elevate in patients with AIS compared with healthy volunteers, suggesting its potential as a novel therapeutic strategy. A miRNA-494 level greater than 1.057 in peripheral blood lymphocytes may help predict an excellent outcome in patients with AIS aged under 65 years, particularly those who do not receive intravenous thrombolysis or endovascular thrombectomy. Additionally, a high level of miRNA-494 or a level greater than 0.858 may indicate minor-to-moderate stroke at admission.

## Availability of Data and Materials

All data used for this study are provided in the manuscript. Additional details are available from the corresponding author on request.

## Supplementary Material

Supplementary material associated with this article can be found, in the online version, at https://doi.org/10.31083/RN37809.
