# Supplementary figures and images for: miRNA-494 in Lymphocytes: A Promising Biomarker for Acute Ischemic Stroke
Source: Rev Neurol. 2025 Aug 29;80(7):37809. doi: 10.31083/RN37809 (PMC12415885; doi:10.31083/RN37809)

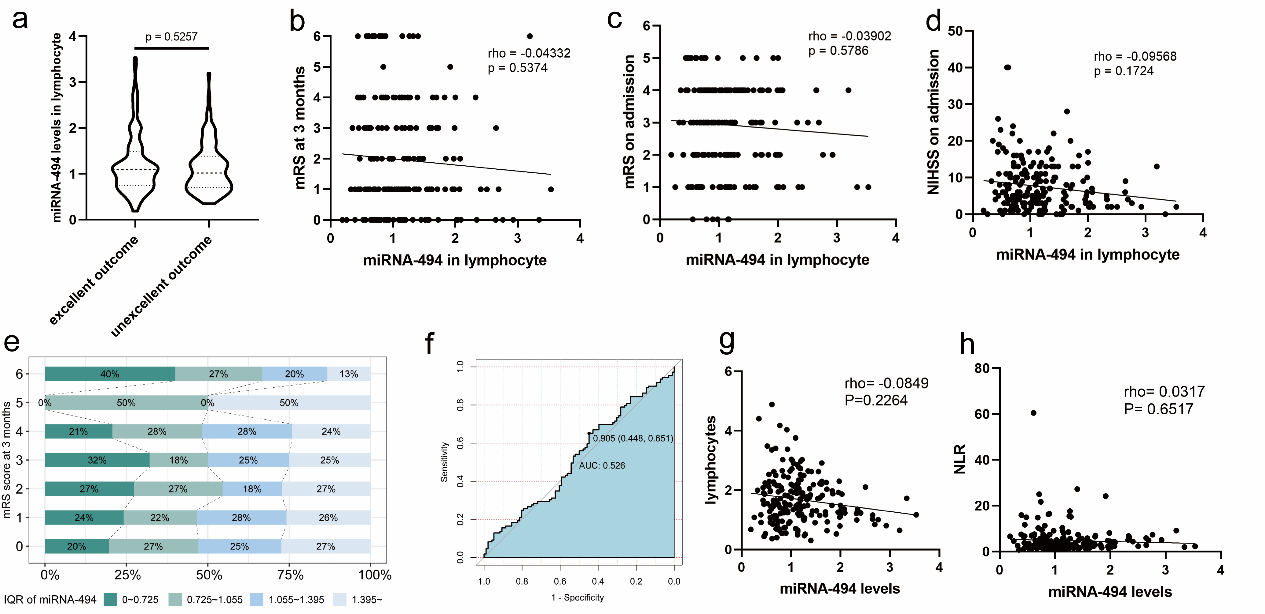


**Supplementary Fig. 1. Analysis between miRNA-494 expression and clinical parameters.**

Supplement: Supplementary file 1 [file 1576-6578-80-7-37809-s1.docx]
